# Nicotine Impairs the Anti-Contractile Function of Perivascular Adipose Tissue by Inhibiting the PPARγ–Adiponectin–AdipoR1 Axis

**DOI:** 10.3390/ijms242015100

**Published:** 2023-10-12

**Authors:** Afifah Zahirah Abd Rami, Amilia Aminuddin, Adila A. Hamid, Mohd Helmy Mokhtar, Azizah Ugusman

**Affiliations:** Department of Physiology, Faculty of Medicine, Universiti Kebangsaan Malaysia, Jalan Yaacob Latif, Cheras, Kuala Lumpur 56000, Malaysia; afifahzahirahabdrami@gmail.com (A.Z.A.R.); adilahamid@ppukm.ukm.edu.my (A.A.H.); helmy@ukm.edu.my (M.H.M.)

**Keywords:** adiponectin, anti-contractile, nicotine, perivascular adipose tissue, PPARγ

## Abstract

Nicotine is an addictive compound found in cigarette smoke that leads to vascular dysfunction and cardiovascular diseases. Perivascular adipose tissue (PVAT) exerts an anti-contractile effect on the underlying vasculature through the production of adipokines, such as adiponectin, which acts on adiponectin receptors 1 (adipoR1) to cause vasorelaxation. Peroxisome proliferator-activated receptor gamma (PPARγ) is a transcription factor that regulates adiponectin gene expression and PVAT development. This study aimed to determine the effect of nicotine on the anti-contractile function of PVAT via the PPARγ–adiponectin–adipoR1 axis. Male Sprague Dawley rats were divided into a control group (given normal saline), a nicotine group (given 0.8 mg/kg of nicotine), and a nicotine + PPARγ agonist group (given nicotine and 5 mg/kg of telmisartan). Thoracic aorta PVAT was harvested after 21 days of treatment. The results showed that nicotine reduced the anti-contractile effect of PVAT on the underlying thoracic aorta. Nicotine also decreased the gene and protein expression of PPARγ, adiponectin, and adipoR1 in PVAT. Treatment with telmisartan restored the anti-contractile effect of PVAT and increased the gene and protein expression of PPARγ, adiponectin, and adipoR1 in PVAT. In conclusion, nicotine attenuates the anti-contractile function of PVAT through inhibition of the PPARγ–adiponectin–adipoR1 axis.

## 1. Introduction

Cardiovascular diseases (CVD) such as coronary artery disease (CAD), stroke, and peripheral artery disease are the leading causes of death worldwide [1]. The pathogenesis of CVD is associated with the formation of an atherosclerotic plaque that narrows blood vessels and reduces blood supply to organs. Smoking is a risk factor for CVD and atherosclerosis. Every year, an estimated 1.9 million deaths worldwide from CVD are attributed to smoking [2]. In addition to causing direct effects on active smokers, passive smokers who are exposed to cigarette smoke in the environment also have an increased risk of CVD [3].

Smoking has become a habit in the community, which commonly starts during adolescence and continues due to the addiction effect [4]. Cigarette smoke contains a mixture of more than 9000 harmful chemicals. Nicotine is one of the most toxic substances in cigarette smoke, in addition to causing addiction [5]. In addition, it has been proven that nicotine has adverse effects on the structure and function of blood vessels. Nicotine triggers oxidative stress that causes vascular endothelial dysfunction. This leads to vasoconstriction, arterial stiffness, and hypertension, which in turn leads to CVD [6].

The conventional understanding of adipose tissues encompasses three distinct types based on their structure, function, and location [7]. White adipose tissue (WAT), primarily found in the hypodermis and around internal organs, primarily consists of cells containing single large lipid droplets. Brown adipose tissue (BAT), which is temporarily present in specific regions in humans such as the interscapular and mediastinal areas, plays a role in maintaining consistent body temperature. It contains adipocytes with numerous cytoplasmic inclusions of varying sizes and is richly vascularized [8]. Beige adipose tissue can be interspersed among WAT cells and has the ability to adopt a brown-like phenotype [7].

In 1991, a new category of adipose tissue, known as perivascular adipose tissue (PVAT), was introduced by Soltis and Cassis. This type of adipose tissue was identified as a neurohumoral regulator of vascular responsiveness [9]. Extensive evidence indicates that PVAT depots exhibit varying phenotypic and functional distinctions based on their location within the vascular bed. For instance, PVAT surrounding the thoracic aorta possesses characteristics akin to BAT, whereas PVAT around the abdominal aorta exhibits characteristics resembling WAT. Conversely, PVAT around mesenteric vessels resembles WAT, while PVAT linked to renal vessels comprises a mixture of white and brown adipocytes [10]. This implies that PVAT may display distinct characteristics depending on its location within the vascular system.

PVAT can be found surrounding the systemic blood vessels, except for the cerebral vessels [11]. Initially, PVAT was believed to function only to support the structure of blood vessels. However, PVAT has been identified as an important endocrine and paracrine organ. PVAT produces various adipokines, cytokines, and other vasoactive factors involved in the regulation of vascular functions. PVAT plays a role in regulating the contractility of the surrounding blood vessels through the secretion of adipocytokines [12]. Under normal conditions, PVAT has an anti-contractile effect on the vasculature [13] through the production of various vasodilators such as adiponectin, angiotensin 1–7, methyl palmitate, and nitric oxide (NO) [14]. The anti-contractile effect of PVAT is impaired in patients with hypertension and metabolic syndrome [10]. 

Adiponectin is the main adipokine that contributes to the anti-contractile function of PVAT. Adiponectin acts by binding to adiponectin receptor 1 in adipocytes, endothelial cells, and vascular smooth muscle cells (VSMC) [15,16]. Adiponectin increases the phosphorylation of endothelial nitric oxide synthase (eNOS), which stimulates the rate of endothelial NO synthesis. NO inhibits VSMC activity to cause vasorelaxation. In addition, adiponectin inhibits the activity of nicotinamide adenine dinucleotide phosphate (NADPH) oxidase. This reduces oxidative stress and preserves the bioavailability of NO [17].

Studies on the effect of smoking on adiponectin levels in the sera of smokers have shown that smokers have low serum adiponectin levels [18]. Interestingly, adiponectin levels in smokers improved after four months of smoking cessation [19]. Nicotine was found to reduce the mRNA expression and secretion of adiponectin in cultured adipocytes [20]. Furthermore, prenatal exposure of rats to nicotine causes obesity and impaired vasodilation during adulthood [21]. Rats exposed to nicotine in utero also showed reduced vasodilation and increased blood pressure [22]. These changes in vasodilation and blood pressure are associated with PVAT dysfunction [21,22].

Peroxisome proliferator-activated receptor gamma (PPARγ) is a transcription factor that regulates the gene expression, development, and differentiation of PVAT. Activation of PPARγ stimulates the production of adiponectin [23]. Moreover, deletion of the PPARγ gene leads to impaired PVAT development, which increases the risk of atherosclerosis [24]. Telmisartan is an angiotensin II receptor antagonist (ARB) commonly used for hypertension treatment. ARBs block angiotensin II type 1 receptors to reduce vasoconstriction, thereby lowering vascular resistance and blood pressure [25]. Additionally, telmisartan acts as an agonist to PPARγ, stimulating adiponectin gene expression and secretion in mouse white adipose tissue [26]. Activation of PPARγ by telmisartan has anti-inflammatory effects within adipose tissue [27]. By enhancing adiponectin levels and activating PPARγ, telmisartan may also contribute to improved insulin sensitivity [28]. On the other hand, sodium nitroprusside (SNP) is a potent non-endothelium-dependent vasodilator and antihypertensive drug. SNP serves as a NO donor, directly acting on VSMC to induce vasorelaxation without relying on NO produced by endothelial cells. In vascular studies, SNP is a valuable tool to explore vascular reactivity and investigate how blood vessels respond to various stimuli [29].

Even though nicotine has been shown to reduce adiponectin and impair vasodilation, the effect of nicotine on the anti-contractile function of PVAT through the PPARγ–adiponectin–adiponectin receptors axis is not fully understood. We hypothesized that nicotine impaired the anti-contractile function of PVAT by inhibiting the PPARγ–adiponectin–adipoR1 axis. Findings from this study may increase our understanding of the mechanism of nicotine-induced vascular dysfunction, and open new opportunities to produce a new therapeutic target for vascular dysfunction due to exposure to nicotine.

## 2. Results

### 2.1. Effect of Nicotine on the Vasoreactive Function of PVAT in the Rat Aorta

#### 2.1.1. Aortic Contraction to Phenylephrine (PE)

**Figure 1:**
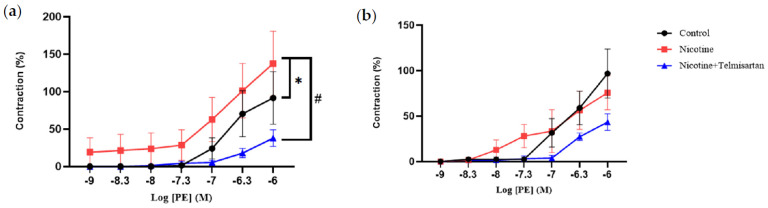
Aortic contraction in response to phenylephrine (PE) in (**a**) PVAT-intact and (**b**) PVAT-removed aortic rings. Values are mean ± SEM, n = 6. * *p* < 0.05 vs. control group, ^#^ *p* < 0.05 vs. nicotine group.

Figure 1a shows the contraction response of aortic rings with PVAT to PE at concentrations ranging from 10^−9^–10^−6^ M. There was a significant increase in the aortic contraction response to PE in rats induced with nicotine (137.55 ± 43.17%) compared with the control rats (91.58 ± 34.94%) (*p* < 0.05). Treatment of nicotine-induced rats with telmisartan decreased the aortic contraction response (37.95 ± 11.04%) compared with the untreated nicotine group (*p* < 0.05). Meanwhile, the PVAT-removed aortic rings did not show any significant difference in their contraction to PE in any of the groups (Figure 1b).

#### 2.1.2. Aortic Relaxation to Sodium Nitroprusside (SNP)

**Figure 2:**
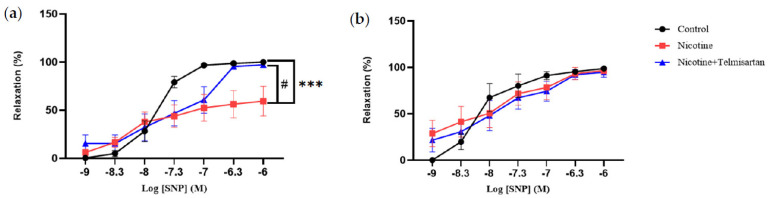
Aortic relaxation in response to sodium nitroprusside (SNP) in (**a**) PVAT-intact and (**b**) PVAT-removed aortic rings. Values are mean ± SEM, n = 6. *** *p* < 0.001 vs. control group, ^#^ *p* < 0.05 vs. nicotine group.

Figure 2a shows the relaxation response of PVAT-intact aortic rings to SNP at concentrations ranging from 10^−9^–10^−6^ M. There was a decrease in the aortic relaxation response to SNP in the nicotine-induced rats (59.46 ± 15.43%) compared with the control group (100.00 ± 0.00%) (*p* < 0.001). Treatment with telmisartan increased the aortic relaxation response to SNP (97.22 ± 2.78%) compared with the untreated nicotine group (*p* < 0.05). The aortic rings without PVAT did not show any significant difference in relaxation response to SNP in any of the groups (Figure 2b).

### 2.2. Effect of Nicotine on Gene and Protein Expression of PPARγ in PVAT

**Figure 3:**
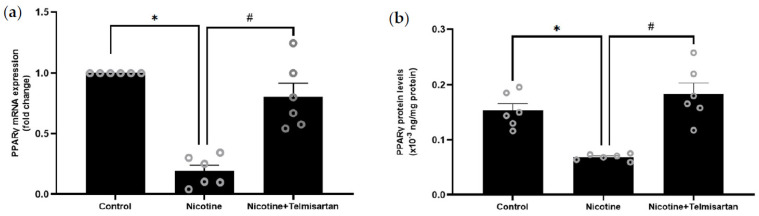
(**a**) PPARγ gene expression and (**b**) PPARγ protein levels in PVAT. Data presented as mean ± SEM, n = 6. * *p* < 0.05 vs. control group, ^#^ *p* < 0.05 vs. nicotine group.

Figure 3a shows the expression of the PPARγ gene in rat PVAT. PPARγ gene expression in the PVAT of nicotine-induced rats was downregulated by 8.33-fold compared with the control rats (*p* < 0.05). Treatment with telmisartan upregulated PPARγ gene expression in the rat PVAT by 6.58-fold compared with the untreated nicotine group (*p* < 0.05). There was no significant difference in PPARγ gene expression between the telmisartan-treated and control groups. Meanwhile, Figure 3b shows the level of PPARγ protein in rat PVAT. There was a decrease in the PPARγ protein levels in the PVAT of the nicotine-induced rats (0.07 ± 0.00 × 10^−3^ ng/mg protein) compared with the control rats (0.16 ± 0.02 × 10^−3^ ng/mg protein) (*p* < 0.05). The PPARγ protein levels in the PVAT of the telmisartan-treated group were increased (0.18 ± 0.04 × 10^−3^ ng/mg protein) compared with the untreated nicotine group (*p* < 0.05). There was no significant difference in PPARγ protein levels between the telmisartan-treated and control groups.

### 2.3. Effect of Nicotine on Gene and Protein Expression of Adiponectin in PVAT

**Figure 4:**
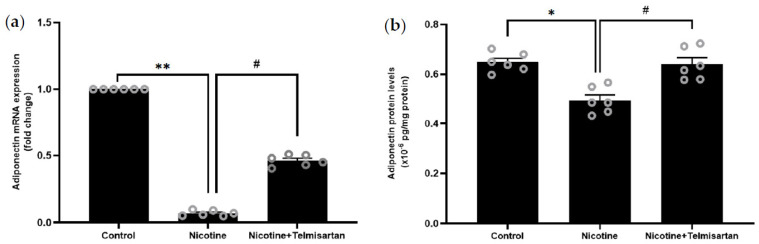
(**a**) Adiponectin gene expression and (**b**) adiponectin protein levels in PVAT. Data presented as mean ± SEM, n = 6. * *p* < 0.05 vs. control group, ** *p* < 0.01 vs. control group, ^#^ *p* < 0.05 vs. nicotine group.

Figure 4a shows the gene expression of adiponectin in rat PVAT. Nicotine downregulated adiponectin gene expression in the rat PVAT by 14.29-fold compared with the control rats (*p* < 0.01). Treatment with telmisartan upregulated adiponectin gene expression in the PVAT by 8.29-fold compared with the nicotine group (*p* < 0.05). There was no significant difference in adiponectin gene expression between the telmisartan-treated and control groups. Figure 4b shows the protein level of adiponectin in the supernatant of homogenized PVAT samples. There was a decrease in adiponectin protein levels in the PVAT of rats induced with nicotine (0.49 ± 0.03 × 10^−6^ pg/mg protein) compared with the control rats (0.64 ± 0.02 × 10^−6^ pg/mg protein) (*p* < 0.05). Meanwhile, there was an increase in adiponectin protein levels in the PVAT with telmisartan treatment (0.64 ± 0.03 × 10^−6^ pg/mg protein) compared with the untreated nicotine group (*p* < 0.05). There was no significant difference in adiponectin protein levels between the telmisartan-treated and control groups.

### 2.4. Effect of Nicotine on Gene Expression and Protein Distribution of AdipoR1 in PVAT 

**Figure 5:**
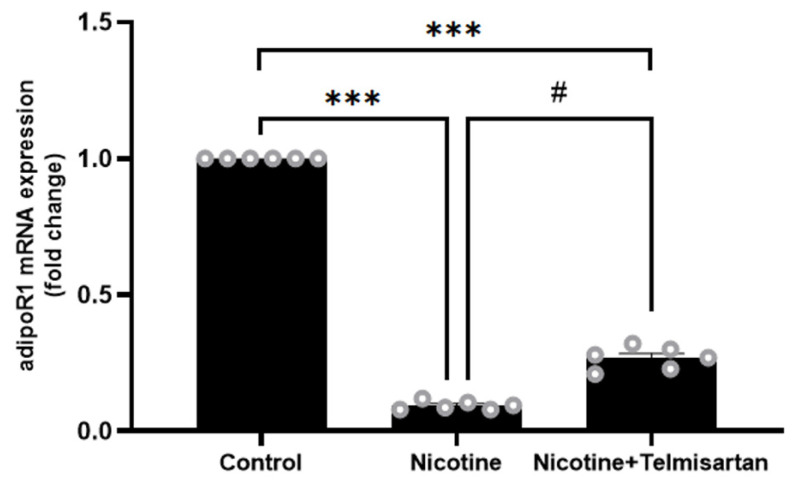
AdipoR1 gene expression in PVAT. Data presented as mean ± SEM, n = 6. *** *p* < 0.001 vs. control group, ^#^ *p* < 0.05 vs. nicotine group.

Figure 5 shows the gene expression of adipoR1 in the rat PVAT. Nicotine downregulated adipoR1 gene expression in PVAT by 11.11-fold compared with the control group (*p* < 0.001). Treatment with telmisartan increased adipoR1 gene expression in PVAT by 2.78-fold compared with the nicotine group (*p* < 0.05). However, the expression of the adipoR1 gene in the group treated with telmisartan was still four times lower than that in the control group (*p* < 0.001).

**Figure 6:**
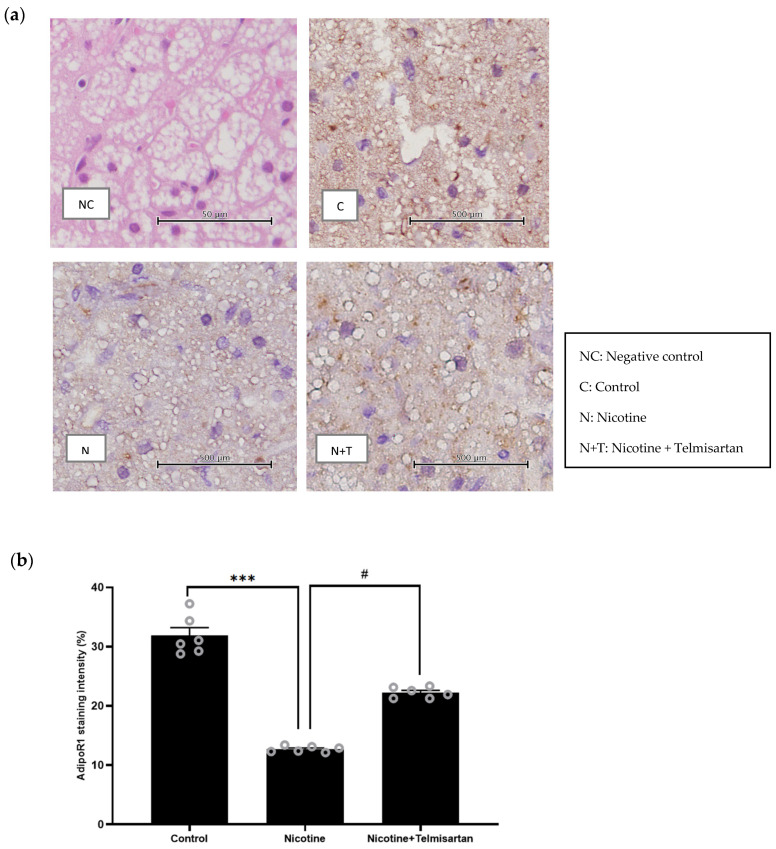
(**a**) Representative images of adipoR1 protein distribution in PVAT (40× objective, scale bar: 50 µm and 500 µm). (**b**) Semi-quantitative analysis of adipoR1 staining intensity in PVAT. Data presented as mean ± SEM, n = 6. *** *p* < 0.001 vs. control group, ^#^ *p* < 0.05 vs. nicotine group. C, control; N, nicotine; NC, negative control; N + T, nicotine + telmisartan.

Figure 6a shows representative images of immunohistochemistry (IHC) staining for adipoR1 protein distribution in PVAT. The negative control (NC) refers to the control PVAT without adipoR1 primary antibody. Positive diaminobenzidine (DAB) staining confirmed the presence of adipoR1 in the adipocytes of the control (C), nicotine (N), and nicotine + telmisartan (N + T) groups. As seen in Figure 6b, adipoR1 staining intensity was significantly reduced in PVAT from the nicotine group compared with the control group (12.70 ± 0.21% vs. 31.85 ± 1.34%, *p* < 0.001). Meanwhile, there was an increase in adipoR1 staining intensity with telmisartan treatment compared with the untreated nicotine group (22.24 ± 0.37% vs. 12.70 ± 0.21%, *p* < 0.05). There was no significant difference in adipoR1 staining intensity between the control and telmisartan-treated groups.

## 3. Discussion

Collectively, our study showed that nicotine impaired the anti-contractile effect of PVAT in rats, most likely by inhibiting the PPARγ–adiponectin–adipoR1 axis. We demonstrated that in PVAT-intact aortic rings, the contractile responses of the thoracic aorta to PE were significantly increased, whereas the non-endothelium-dependent relaxation responses of the thoracic aorta to SNP were significantly decreased in nicotine-induced rats. SNP acts as a NO donor, directly inducing vasorelaxation independently of NO generated by endothelial cells [29]. NO activates soluble guanylate cyclase in VSMC that converts guanosine triphosphate to cyclic guanosine monophosphate (cGMP). cGMP lowers intracellular Ca^2+^ levels and stimulates protein kinase G (PKG). PKG, in turn, activates myosin light chain phosphatase, leading to vasorelaxation [30]. 

Nicotine, through activation of nicotinic acetylcholine receptors, alters calcium signaling in the VSMC, leading to amplified calcium release from the sarcoplasmic reticulum, increased calcium influx from the extracellular space, and heightened calcium sensitivity [31]. These changes result in enhanced vasoconstriction. Moreover, nicotine promotes the formation of superoxide, which degrades NO. The reduction in NO availability diminishes the vasodilatory effects of SNP [6]. Nicotine administration has been shown to increase plasma angiotensin-converting enzyme activity in rats, leading to a higher conversion of angiotensin I to angiotensin II. Nicotine also increases the expression of angiotensin II type 1 receptors in the vasculature, which further amplifies the vasoconstrictive effect of angiotensin II [32]. 

Meanwhile, treatment of nicotine-induced rats with a PPARγ agonist and angiotensin receptor blocker, telmisartan, significantly decreased the contractile responses of the thoracic aorta to PE and enhanced the relaxation responses of the thoracic aorta to SNP. Telmisartan prevents angiotensin II from binding to angiotensin II type 1 receptors in the vasculature. This inhibits the vasoconstrictive effects of angiotensin II, leading to vasorelaxation [33]. Telmisartan also increases the bioavailability of NO [34], thus enhancing vasorelaxation response to SNP. 

The impaired vasorelaxant effect of nicotine was not observed in PVAT-denuded aortic rings. This finding suggests that nicotine results in PVAT dysfunction, which leads to the attenuation of PVAT’s anti-contractile effect. Previously, Gao et al. demonstrated that prenatal exposure to nicotine in rat offspring caused a postnatal decrease in vasorelaxation and an increase in blood pressure, which could be due to PVAT dysfunction [22]. However, Gao et al. did not perform further analysis on the underlying mechanisms of PVAT dysfunction, such as the involvement of the PPARγ–adiponectin–adiponectin receptor axis. Therefore, in this study, we determined the role of PPARγ–adiponectin–adiponectin receptors axis in mediating the loss of the anti-contractile effect of PVAT in rats exposed to nicotine.

Our results showed that nicotine reduced the gene and protein expression of PPARγ, adiponectin, and adipoR1 in the rat PVAT. Meanwhile, treatment with a PPARγ agonist, telmisartan, increased the gene and protein expression of PPARγ, adiponectin, and adipoR1 in the PVAT of nicotine-induced rats. These findings suggest that nicotine impairs the anti-contractile function of PVAT by inhibiting the PPARγ–adiponectin–adipoR1 axis.

PPARγ is a transcription factor that plays an important role in the regulation of gene expression, formation, and differentiation of adipose tissue [35]. Cigarette smoke extracts affect the transcription of various target genes by binding to PPARγ [36]. A previous study also showed that mice exposed to cigarette smoke for three months had low PPARγ gene expression and protein levels in their alveolar macrophages [37]. Additionally, the deletion of the PPARγ gene disrupts PVAT development and reduces mouse aortic vasorelaxation [24]. 

One of the mechanisms that mediates the vasorelaxant effect of PPARγ is through the production of adiponectin. PPARγ is the main regulator of adiponectin expression and secretion [38]. PPARγ enhances the production of adiponectin by stimulating adiponectin gene transcription, adiponectin synthesis, and secretion by adipose tissues [39]. PPARγ stimulates adiponectin gene expression by binding to the PPAR response element of the adiponectin gene promoter [40]. A previous study also showed that PPARγ increases adiponectin secretion by inhibiting the transcription of endoplasmic reticulum chaperone protein 44 (ERp44). ERp44 causes adiponectin to be stored in adipocytes through covalent binding to the thiol groups. Hence, the inhibition of ERp44 by PPARγ increases the secretion of adiponectin from adipocytes [41]. 

PPARγ also stimulates the transport of vesicles containing adiponectin through reptin activation. This accelerates the polymerization and secretion of adiponectin by adipose tissues [23]. Furthermore, treatment of diabetic rats with a PPARγ agonist, telmisartan, increases adiponectin levels [42]. These findings support the results of our study, in which nicotine decreased the PPARγ gene and protein expression, leading to decreased adiponectin gene and protein expression in the PVAT, whereas treatment with telmisartan reversed these effects.

Adiponectin produced by PVAT has an anti-contractile effect on the underlying blood vessels through endothelium-dependent and endothelium-independent mechanisms [43]. Adiponectin’s endothelium-dependent mechanism of vasorelaxation is mediated through the activation of the AMPK–PI3K–Akt–eNOS pathway in the endothelial cells [44]. In this study, we used endothelium-denuded aortic rings. Hence, in our study, adiponectin caused vasorelaxation through an endothelium-independent mechanism by acting directly on the VSMC. Adiponectin stimulates the opening of large-conductance voltage- and Ca^2+^-activated K^+^ (BK_Ca_) channels in VSMC [45]. The opening of BK_Ca_ channels causes K^+^ efflux from VSMC, leading to hyperpolarization and vasorelaxation [46]. 

Adiponectin acts mainly by binding to adipoR1, which is abundant in adipose tissues, endothelial cells, and VSMC [15,16]. In this study, nicotine reduced the gene expression and protein distribution of adipoR1 in PVAT. Loss of the anti-contractile effect of PVAT in obese mice is also associated with reduced adipoR1 protein expression in the PVAT [47]. A previous study showed that smokers have a single nucleotide polymorphism in their adipoR1 gene that causes reduced adipoR1 gene expression [48]. Additionally, nicotine stimulates the ubiquitination of adipoR1, leading to degradation and decreased levels of adipoR1 protein [49]. 

Lowered amounts of the adipoR1 protein lead to a reduction in the binding of adiponectin to it. This results in decreased adiponectin-mediated effects on target tissues [50]. Therefore, in this study, the lack of adiponectin capable of binding to adipoR1 in PVAT resulted in a decrease in the anti-contractile effect of PVAT. A previous study also showed that treatment with telmisartan increased the gene expression and protein distribution of adipoR1 in the coronary arteries of diabetic rats through the activation of PPARγ [42]. These results align with the outcomes of this study, which demonstrated increased gene expression and protein distribution of adipoR1 in the rat PVAT with telmisartan treatment. 

Collectively, apart from demonstrating that nicotine impaired the anti-contractile effect of PVAT by inhibiting the PPARγ–adiponectin–adipoR1 axis, we also showed that telmisartan successfully reversed the negative effects of nicotine on the anti-contractile function of PVAT. Administration of telmisartan to nicotine-induced rats improved vasorelaxation and increased the gene and protein expression of PPARγ, adiponectin, and adipoR1 in PVAT. This suggests that telmisartan can counter the negative effects of nicotine on the anti-contractile function of PVAT by enhancing the PPARγ–adiponectin–adipoR1 pathway. 

Adipocytes constitute the majority of cells in PVAT. Additionally, PVAT comprises a stromal vascular fraction encompassing fibroblasts, mesenchymal stem cells, lymphocytes, macrophages, and the vasa vasorum-lining endothelial cells [10]. Meanwhile, adiponectin is exclusively produced by adipocytes. Hence, the results of the study are most likely contributed by adipocytes in the PVAT. 

However, there are a few limitations to the current study. We only used endothelium-denuded aortic rings because we wanted to focus on the anti-contractile function of PVAT alone without the influence of other vasoactive factors produced by the endothelium. Therefore, the role of endothelium and the AMPK–PI3K–Akt–eNOS pathway in mediating the effect of nicotine on the anti-contractile function of PVAT needs to be explored in future studies. The effect of nicotine and PPARγ agonists on adiponectin receptor signaling in PVAT should also be further investigated. In addition, the outcomes of our study cannot be directly extrapolated beyond the thoracic aorta, given the substantial variations in PVAT structure and composition in different blood vessels within the vascular system. 

## 4. Materials and Methods

### 4.1. Animal and Study Design

The study was approved by the Animal Ethics Committee of Universiti Kebangsaan Malaysia (UKM) (approval code: FISIO/FP/2021/AZIZAH UGUSMAN/24-MAR./1160-APR.-2021-MAR.-2023). Six-week-old male Sprague Dawley rats (180–230 g) were obtained from the Animal Resource Unit of UKM. Each rat was kept in a cage and maintained under standard conditions of a 12 h light/dark cycle. The rats were fed a standard rat chow diet with water ad libitum. The rats were randomly divided into three groups (n = 6 per group): the control group was administered normal saline via intraperitoneal injection (i.p.) and oral gavage (p.o.); the nicotine group was administered 0.8 mg/kg/day nicotine (Tokyo Chemical Industry, Tokyo, Japan) via i.p.; and the nicotine + telmisartan group was administered 0.8 mg/kg/day of nicotine via i.p. and 5 mg/kg/day of telmisartan via p.o. Treatment was given daily for 21 days. The dosage and duration of nicotine treatment mimic the exposure of a chronic light smoker and have been proven to cause vascular dysfunction [51]. The dosage of telmisartan was previously shown to increase the gene expression of PPARγ and adiponectin in rats [26]. On day 22, the rats were terminally anesthetized with an intravenous injection of ketamine and xylazine cocktail (0.2 mL/kg). Thoracic aortae with the PVAT attached were then harvested, and part of the fresh aortic tissues was immediately used for wire myography. The remaining PVAT samples were used for gene and protein analyses.

### 4.2. Wire Myography

The thoracic aortae were cut into 4 mm PVAT-intact and PVAT-removed aortic rings. In all the experiments, the endothelium was removed by gently rubbing the interior of the vessel around a wire. The removal was confirmed by the lack of a vasodilator response to 10^−6^ M acetylcholine (ACh) (Tokyo Chemical Industry, Tokyo, Japan). The aortic rings were mounted on two stainless steel pins in a four-channel wire myograph (Danish Myo Technology, Ann Arbor, MI, USA). The vessels were bathed in physiological Krebs solution with the following composition: 118 mM of NaCl, 4.7 mM of KCl, 11 mM of glucose, 1.2 mM of MgSO_4_, 25 mM of NaHCO_3_, 1.03 mM of KH_2_PO_4_, and 2.5 mM of CaCl_2_. The vessels were then gassed continuously with 95% O_2_ and 5% CO_2_ at 37 °C. The aortic rings were set to an optimum tension of 1 g and allowed to equilibrate for at least 30 min before use [52]. Following calibration, the viability of the aortic rings was tested using 40 mM of KCl. The cumulative contraction responses of the aortic rings to 10^−9^–10^−6^ M of PE (Sigma, Livonia, MI, USA) were then measured. To measure the relaxation response, the rings were pre-contracted with 10^–6^ M of PE. The cumulative relaxation responses to an endothelium-independent vasodilator, SNP (Sigma, USA), were recorded by adding 10^–9^–10^–6^ M of SNP. The PowerLab Data Acquisition System (ADInstruments, Bella Vista, Australia) was used to measure and record changes in vessel tension. 

### 4.3. RNA Extraction and Quantitative Real-Time Polymerase Chain Reaction (qPCR)

Total RNA was extracted from PVAT using the RNeasy Lipid Tissue Mini Kit (Qiagen, Hilden, Germany). RNA extraction was performed according to the manufacturer’s protocol. A Viva cDNA Synthesis Kit (Vivantis, Shah Alam, Malaysia) was used for cDNA synthesis following the protocol provided. The master mix solution was prepared using the ViPrimePLUS Taq qPCR Green Master Mix I (SYBR Green Dye) kit (Vivantis, Shah Alam, Malaysia) in accordance with the kit’s manual, and qPCR was then performed using the Bio-Rad CFX96 Real-Time System. The sequences of the forward and reverse primers used are listed in Table 1. Each analysis was performed in triplicate. Gene expression was calculated using the 2^−ΔΔCt^ equation, as previously described [53]. 

### 4.4. Protein Extraction and Enzyme-Linked Immunosorbent Assay (ELISA)

PVAT samples were homogenized using an Omni Bead Ruptor homogenizer (Perkin Elmer, Waltham, MA, USA). Protein was extracted from the homogenized PVAT using radioimmunoprecipitation assay (RIPA) buffer with protease inhibitor solution. ELISA was then performed using rat PPARγ and adiponectin ELISA kits (Finetest, Wuhan, China) according to the kits’ protocol. The samples were added to a plate coated with rat anti-PPARγ or anti-adiponectin. Subsequently, horseradish peroxidase (HRP)-conjugated avidin and tetramethyl benzidine substrates were added to the plate prior to the stop solution. The optical density of the samples was measured at a wavelength of 450 nm. Total protein levels in the PVAT samples were measured using the bicinchoninic acid assay technique. The protein levels of PPARγ and adiponectin were normalized to the amount of total protein in the samples.

### 4.5. Immunohistochemistry (IHC) Analysis

PVAT samples were fixed in 10% formalin, dehydrated, and embedded in paraffin wax. The PVAT sections were cut on a rotary microtome and used for IHC analysis using a mouse- and rabbit-specific HRP/DAB IHC detection kit (Abcam, Waltham, MA, USA). The slides were first treated with antigen retrieval solution (Dako, Glostrup, Denmark), followed by hydrogen peroxide to inhibit endogenous peroxidase. The samples were then incubated with a protein-blocking serum to prevent excessive background staining. To analyze the distribution of protein, the slides were incubated with a mouse monoclonal antibody against adipoR1 at a 1:500 dilution (Santa Cruz Biotechnology, Dallas, TX, USA). The slides were then incubated with micropolymer secondary antibody, followed by DAB substrate. Subsequently, the slides were counterstained with hematoxylin and sequentially dehydrated. The negative control slides were stained, without incubation, with adipoR1 primary antibody. The sections were photographed using an Olympus SZ61TR-TP051000 microscope (Olympus, Tokyo, Japan), and the images were analyzed using ImageJ software version 8 (ImageJ Software, National Institute of Health, Bethesda, MD, USA).

### 4.6. Data Analysis

GraphPad Prism software version 9 (GraphPad Software, La Jolla, CA, USA) was used for data analysis. The Shapiro–Wilk normality test was used to test the normality of the data distribution. The data obtained are presented in the form of mean ± standard error of the mean (SEM). Differences between the groups were analyzed using the analysis of variance (ANOVA) test followed by Tukey’s post hoc test. Differences were considered statistically significant at a value of *p* ˂ 0.05.

## 5. Conclusions

Exposure to nicotine reduces the gene and protein expression of PPARγ, adiponectin, and adipoR1 in the thoracic aorta PVAT. These alterations are likely to play a role in the loss of the anti-contractile effect of PVAT in rats subjected to nicotine exposure. Therefore, the PPARγ–adiponectin–adipoR1 axis in PVAT may serve as a new target therapy for vascular dysfunction due to nicotine exposure in the future.

## Figures and Tables

**Table 1:** Primer sequences for qPCR analysis.

Gene	Sequence	NCBI Code
Glyceraldehyde 3-phosphate dehydrogenase	Forward: 5′-CTCTCTGCTCCTCCCTGTTC-3′ Reverse: 5′-GGTAACCAGGCGTCCGATAC-3′	NM_017008.4
Adiponectin	Forward: 5′-TCTGGGAGATCCTCCTGTTGA-3′ Reverse: 5′-CGAAGTTGGTGGGCCAGAAT-3′	NM_144744.3
PPARγ	Forward: 5′-CTGGCTCCAAGTGTATGGGG-3′ Reverse: 5′-TTTGATTCTCGGGGCTACGG-3′	NM_013124.3
AdipoR1	Forward: 5′-CTCCCTCCTGGATAGGGTCG-3′, Reverse: 5′-CAGCTTGAGGAGAGGTTGGG-3′	NM_207587.2

## Data Availability

Data can be provided upon request to the corresponding author.
